# Spatial variations and multi-level determinants of modern contraceptive utilization among young women (15–24 years) in Ethiopia: spatial and multi-level analysis of mini-EDHS 2019

**DOI:** 10.1186/s40834-023-00224-0

**Published:** 2023-04-10

**Authors:** Mehari Woldemariam Merid, Anteneh Ayelign Kibret, Adugnaw Zeleke Alem, Melaku Hunie Asratie, Fantu Mamo Aragaw, Dagmawi Chilot, Daniel Gashaneh Belay

**Affiliations:** 1 Department of Epidemiology and Biostatistics, Institute of Public Health, College of Medicine and Health Sciences, University of Gondar, Gondar, Ethiopia; 2 Department of Human Anatomy, College of Medicine and Health Sciences, University of Gondar, Gondar, Ethiopia; 3 Department of Women’s and Family Health, School of Midwifery, College of Medicine and Health Sciences, University of Gondar, Gondar, Ethiopia; 4 Department of Human Physiology, College of Medicine and Health Sciences, University of Gondar, Gondar, Ethiopia

**Keywords:** Spatial variation, Associates, Modern contraceptive, Young women, Ethiopia

## Abstract

**Introduction:**

There is tremendous regional inequalities and low uptake of modern contraceptives particularly among young women (15–24 years), characterized by high fertility but high unmet need for contraceptives in Ethiopia. Hence, the present study aimed at exploring the spatial distribution and the multi-level determinants of modern contraceptive use among young women in Ethiopia.

**Methods:**

This study was conducted using the 2019 Ethiopian demographic and health survey data on a weighted sample of 3,379 young women. STATA version 14 for the multi-level, and ArcGIS 10.7 and Sat Scan 9.6 for the spatial analysis were used. Spatial analysis was done to identify the hotspot areas of modern contraceptive use in Ethiopia. Multi-variable multi-level logistic regression was used for identifying determinants of modern contraceptive use and variables with a *p*-value < 0.05 were considered to be significant determinants.

**Result:**

The overall prevalence of modern contraceptive use among young women in Ethiopia was 17.23% (95% CI: 10.98, 23.47). The hotspots areas for modern contraceptive use were detected in the central and south-western Amhara, western and central Oromia, and western SNNPR regions. Whereas the Somali region, Dire dawa, and Harari cities were cold spot areas for modern contraceptive use.

Being married (AOR = 18.5; 95% CI: 12.66, 27.27), parity (AOR = 4.82; 95% CI: 1.27, 18.32), having television (AOR = 2.39; 95%CI: 1.43, 3.99), having radio (AOR = 1.43; 95%CI: 1.05, 1.94) had higher odds of using modern contraceptives compared to their counterparts. Besides, family size of above five (AOR = 0.46; 95% CI: 0.34, 0.62) and living in Somali region (AOR = 0.05; 95% CI: 0.01, 0.32) were associated with decreased odds of using modern contraceptives among young women in Ethiopia.

**Conclusion:**

The modern contraceptive use was low among young women and considerably varied across regions in Ethiopia. A remarkably low rate of modern contraceptive use (cold spot) area was detected in Somali region-Ethiopia. Taking in to account a geographic perspective and key factors identified in this study would be vital for efficient resource allocation, targeted interventions, and informed decision-making to enhance contraceptive uptake in Ethiopia.

## Background

The United Nations Department of Economic and Social Affairs (DESA) press release on 15^th^ of November 2022 notified the world to celebrate the birth day of the 8th billion person in the planet [1]. The report has announced that the world population could grow to around 9.7 billion in 2030 and 10.4 billion in 2050. In this regard, Africa is expected to account for more than half of the world population growth between 2015 and 2050 and Ethiopia is one of the 8 countries with highest fertility rate and population growth worldwide. The fertility rate is one of the key determinant factors in human development index affecting life expectancy, education, per capita and other indicators [2]. The role of modern contraceptives in decreasing maternal and child morbidity and mortality, and related health costs is indispensable [3, 4].

A large scale demographic and health survey conducted among the six South Asian countries showed that the prevalence of prevalence of modern contraceptive use ranged between 11.9% in India and 28.4% in Bangladesh, where the overall prevalence was is 19.1% [5]

Between 1990 and 2014, the rate of modern contraceptive use among women in most sub-Saharan Africa countries has been increasing in line with global trends [6]. In countries such as Zambia, Lesotho, Zimbabwe, there has been a dramatic increment (above 50%) in the modern contraceptives utilization rate among women of reproductive age group. Despite such improvements in the uptake of modern contraceptives in Africa, the overall prevalence of modern contraceptive use remains low. For instance, a recent representative study reported that the overall prevalence of modern contraceptive use in Africa between 2012 and 2017 was 23% [7]. Another large scale population study in 20 African countries also noted low prevalence of modern contraceptive use (26%) with significant variations across countries, 6% in Guinea and 62% in Zimbabwe [8].

In Ethiopia, the prevalence of any modern contraceptive use by currently married Ethiopian women has steadily increased from 14% in 2005 to 41% in 2019 [9]. The injectables (27%) and implants (9%) were the most commonly used contraceptive methods among currently married women. Sub-national inequalities in modern contraceptives use was also observed where the lowest contraceptive utilization was located in the Somali region (3%) and the highest from Oromia region (50%) and Amhara (49.5%) regions of Ethiopia [9].

Different literatures have identified a number of individual and community level factors that could positively or negatively affect the use of modern contraceptive methods among women of reproductive age. These factors include maternal age, educational status, wealth index, marital status, births in the last three years, women autonomy, partner/husband influence, knowledge on contraceptives, religion, region, and place of residence, media exposure, number of desired children, and family size [10–22].

Nonetheless, the 2020 health sector transformation plan (HSTP) II performance report noted that the national target (55%) contraceptive prevalence rate was not achieved [23]. Particularly, the unmet need for modern contraceptives was much higher among the young women (15–24 years) and half of all pregnancies were unintended compared to the all women of reproductive age in low and middle-income countries including Ethiopia [24]. The Ethiopian mini-DHS 2019 report has also indicated a lower modern contraceptive utilization rate among the young women [9]. Hence, to achieve the HSTP-II national target of reaching 50% contraceptive prevalence rate (CPR) by 2024/25 [23], it is imperative to meet the needs of the young women, characterized by high fertility age group, key productive population segment, but high unmet need for contraceptives and highly vulnerable in someway [9, 19]. Therefore, the present study aimed at exploring the spatial distribution and the multi-level determinants of modern contraceptive use among young women in Ethiopia using the most recent national survey, mini- EDHS 2019 data.

## Method

### Data sources and populations

The data used for the present study was obtained from the 2019 Ethiopian Mini Demographic and Health Survey (EMDHS) dataset, the fifth DHS implemented in Ethiopia from March 21, 2019 to June 28, 2019. The 2019 EMDHS was conducted by the Central Statistical Agency in partnership with the Federal Ministry of Health (FMoH) and the Ethiopian Public Health Institute.

The sample used for the survey was stratified and selected using two stages. Firstly, a total of 305 EAs (93 in urban, 212 in rural) were chosen independently with a probability proportional to each EAs. Second, from the newly formed household listing, a fixed number of 30 households/clusters were selected with an equal probability of systematic selection. The detailed sampling procedures are available on the measure DHS website in the 2019 EMDHS report accessible on (https://www.dhsprogram.com). Data were obtained from the DHS website by justifying the reason for requesting the data and after obtaining an approval letter from the DHS. The IR (individual record) data set was used. In this study, all the women aged 15–24 years living in the selected EAs were the study population. A total weighted sample of 3,379 women aged 15–24 years were included.

### Variables of the study

#### Outcome variable

The use of modern contraceptive was the outcome variable of the study. A woman was considered as a “modern contraceptive user” if she had been utilizing any modern contraceptive methods such as oral contraceptives, male and female sterilization, intrauterine contraceptive device, injectables, implants, male and female condoms, lactational amenorrhea method, standard days method, and emergency contraception [25] during the 2019 EMDHS survey period while woman who had been utilizing traditional, folkloric or no method was considered as a “non-utilizer of modern contraceptive”.

#### Independent variables

Both the individual and community level explanatory variables were used to assess modern contraceptives utilization among women aged 15–24 years.

#### Individual level factors

Current age of the women, highest educational level attained, marital status, household wealth index, total children ever born, number of living children, family size, births in the last three years, family size, presence of son/daughter in the household, possession of TV/radio in the household, and women’s knowledge on modern contraceptive method were the individual level factors considered for the study.

#### Community level variables

Community level independent variables include religion, region, and place of residence, community level education and wealth-index. The community wealth status and literacy levels were categorize as high or low, and the median value was used as the cut-off point for classification since the data was not normally distributed. The community wealth level was classified as high if the proportion of women from the two lowest wealth quintile in a given community was greater than the median value and low if the proportion was below the median value [26]. The community level of women's education was the proportion of women in the community with at least a primary level of education, classified as high (proportion of women greater than median national value) whereas low (proportion of women below-median national value) [27].

### Data management and analysis

Data extraction, editing, coding, and cleaning were performed using STATA 14. The data was weighted using sampling weight (v005) to restore the survey's representativeness and obtain valid statistical estimates. We used a multilevel logistic regression analysis by assuming that each community has a different intercept and fixed coefficient, the clustered data nature as well as within and between community variations, with a random effect applied at the cluster level. Variables with *p*-values ≤ 0.2 in the bi-variable analysis were fitted in the multivariable model. Adjusted Odds Ratio (AOR) with a 95% Confidence Interval (CI) and *p*-value < 0.05 in the multivariable model were used to declare a significant association with the outcome. The goodness of fit was checked using the deviance. As such, the best fitted model (model-III) was used to assess the factors associated with use of modern contraceptive among young women in Ethiopia. After controlling for the confounding effect, marital status, family size, parity, presence of daughter at home, house has TV, household has radio, and Somali region were the variables which remained significantly associated with the use of modern contraceptives.

### Spatial analysis

Spatial analysis was conducted using the Geographic Information System (GIS) application to assess geographic variations of modern contraceptive use among clusters in the 2019 mini EDHS. We received the GPS points in shape file format for the 2019 EDHS survey from the DHS office upon request. We calculated the proportions of modern contraceptive users for each cluster in the survey and then appended the latitude and longitude coordinates of the selected enumeration areas (EAs) in the 2019 EDHS survey. The spatial autocorrelation statistic (Global Moran's I) was used to determine whether modern contraceptive use was dispersed, clustered, or randomly distributed in Ethiopia.

The Getis-Ord Gi* hot spot analysis was used to identify clusters of high values (hot spot) and of low values (cold spot) of modern contraceptive use among young women in Ethiopia. To determine the statistical significance of clustering, the z-score with a *p*-value was computed. A positive z-score with a pP-value of 0.05 indicates clustering of statistically high hotspots; however, a negative z-score with a *P*-value of 0.05 indicates clustering of statistically low cold spots. A z-score close to zero indicates that there is no significant clustering.

The spatial interpolation technique is used to predict modern contraceptive use for the unsampled areas based on sampled clusters. The geostatistical ordinary Kriging spatial interpolation technique was employed for the prediction of unsampled clusters. The interpolation was done based on the assumption that spatially distributed objects are spatially correlated; in other words, things that are close together tend to have similar characteristics [28, 29].

Bernoulli-based model spatial scan statistics were used to determine the geographical locations of statistically significant clusters for modern contraceptive use among women in Ethiopia. A likelihood ratio test statistic and the p-value were used for each potential cluster to determine whether the number of observed modern contraceptive use among women within the potential cluster was significantly higher than expected or not.

## Result

### Socio-demographic characteristics of the women

A total of weighted 3,379 young (15–24) years women were included in the study. More than half (61.5%) of the women were aged 19 to 20 years and 2203 (65.18%) were rural residents. Two-third (68.69%) of the women were married. Regarding the household wealth status, 501 (15%) and 951 (28%) of the women were from poorest and richest households, respectively. While only 7% of the women had attained higher education, the remaining had secondary and lower level of education. More than three-fourth (77.96%) of the women had given birth in the past three years (Table 1).

**Table 1:** Socio-demographic characteristics of young women in Ethiopia, 2022 (3379)

**Variables**	**Categories**	**Weighted Frequency (%)**
**Individual level factors**		
**Current age of women in years**	15–19	2087 (61.5)
	20–24	1292 (38.5)
**Highest educational level**	no education	462 (13.00)
	primary	2012 (60.0)
	secondary	667 (20.0)
	higher	238 (7.0)
**Marital status**	married	1138 (33.67)
	Not married	2241 (66.33)
**Wealth status**	poorest	501 (15.0)
	poorer	584 (17.0)
	middle	606 (18.0)
	richer	738 (22.0)
	richest	951 (28.0)
**Family size**	Less than 5	1390 (41.13)
	5 and above	1989 (58.87)
**Parity**	None	2447 (72)
	One and above	932 (28)
**Number of living children**	None	2467 (73)
	One and above	912 (27)
**Women gave birth in past 3 years**	None	2634 (77.96)
	One and above	745 (22.04)
**Son (s) present at home**	None	569 (16.84)
	One and above	2810 (83.16)
**Daughter (s) present at home**	None	511 (15.13)
	One and above	2,868 (84.87)
**Contraceptive knowledge**	Yes	3175 (93.97)
	No	204 (6.03)
**Household has TV**	Yes	658 (19.48)
	No	2720 (80.52)
**Household has radio**	Yes	966 (28.6)
	No	2413 (71.40)
***Community level factors***		
**Place of residence**	Urban	1176 (34.81)
	Rural	2203 (65.18)
**Region**	Tigray	231 (6.85)
	Afar	30 (0.88)
	Amhara	755 (22.34)
	Oromia	1341 (39.69)
	Somali	160 (4.75)
	Benishangul	37 (1.10)
	SNNPR	595 (17.6)
	Gambela	15 (0.46)
	Harari	10 (0.29)
	Addis Ababa	177 (5.24)
	Dire Dawa	27 (0.80)
**Proportion of Muslims in the cluster**	High	1157 (34.25)
	Low	2222 (65.75)
**Community-level education**	High	1985 (58.74)
	Low	1394 (41.26)
**Community-level wealth**	High	1620 (47.93)
	Low	1759 (52.07)

### Prevalence of modern contraceptive use among young women

**Fig. 1:**
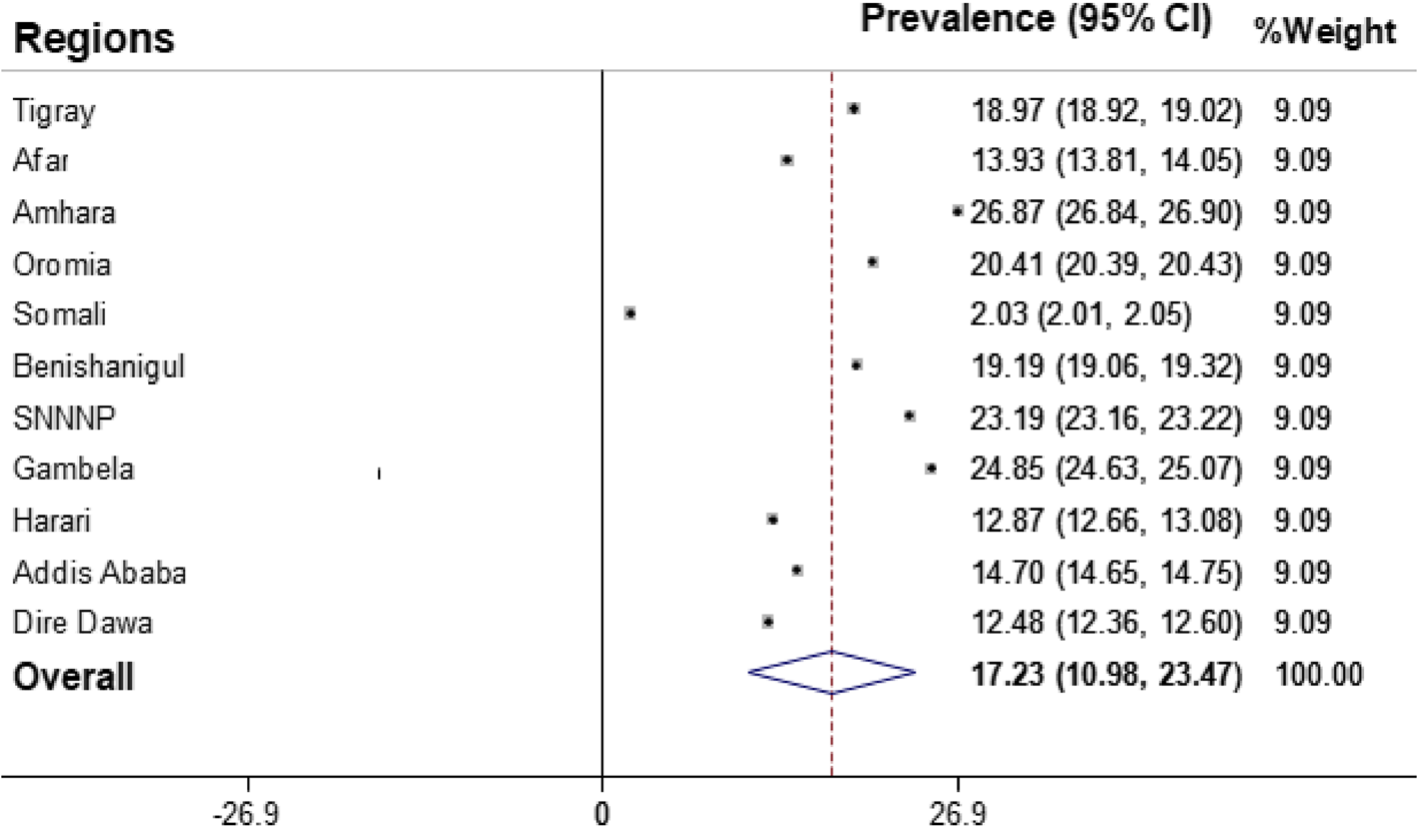
Prevalence of modern contraceptive use among young women in Ethiopia, 2022 (3,379)

In this study, the overallprevalence of modern contraceptive use among young women in Ethiopia was 17.23% (95% CI: 10.98, 23.47). The higher proportions (26.87%) of young women in Amhara region use modern contraceptives whereas lower proportion (2.03%) of women use it in Somali region (Fig. 1).

### Spatial distribution of modern contraceptive use

**Fig. 2:**
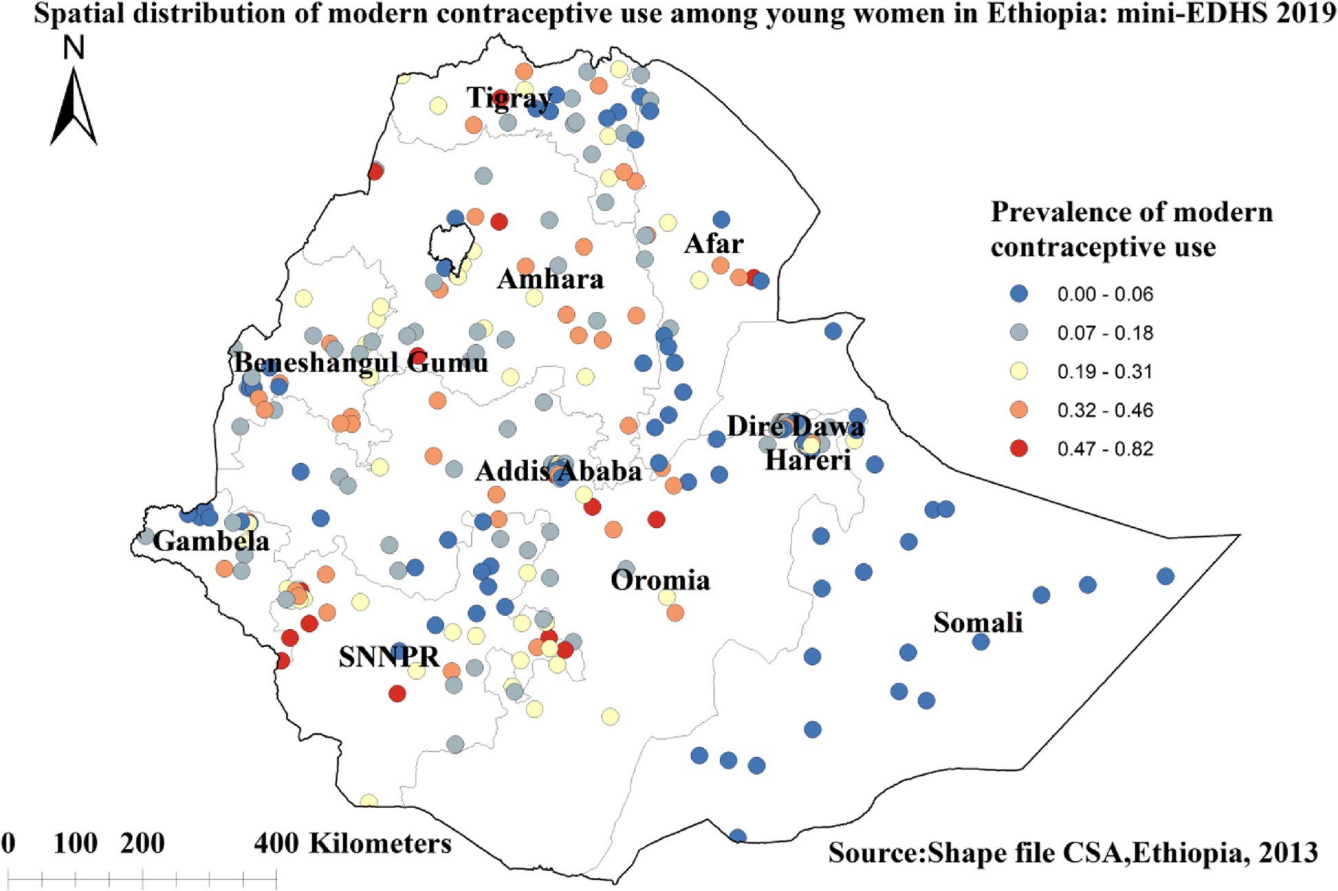
Spatial distribution of modern contraceptive use among young women in Ethiopia: mini EDHS 2019

In Ethiopia, the higher proportion of modern contraceptive use among young women was located in western Tigray, central parts of Amhara region, northern parts of Oromia, western SNNPR, some parts of Addis Ababa. Whereas the lower proportion of modern contraceptive use was observed in the entire Somali region, southern Amhara, central and eastern parts of Tigray, some parts of Benishangul-Gumuz, in most parts of Dire Dawa and Harari cities, in most parts of Gambella, and southern Afar regions (Fig. 2).

### Spatial autocorrelation of modern contraceptive use

**Fig. 3:**
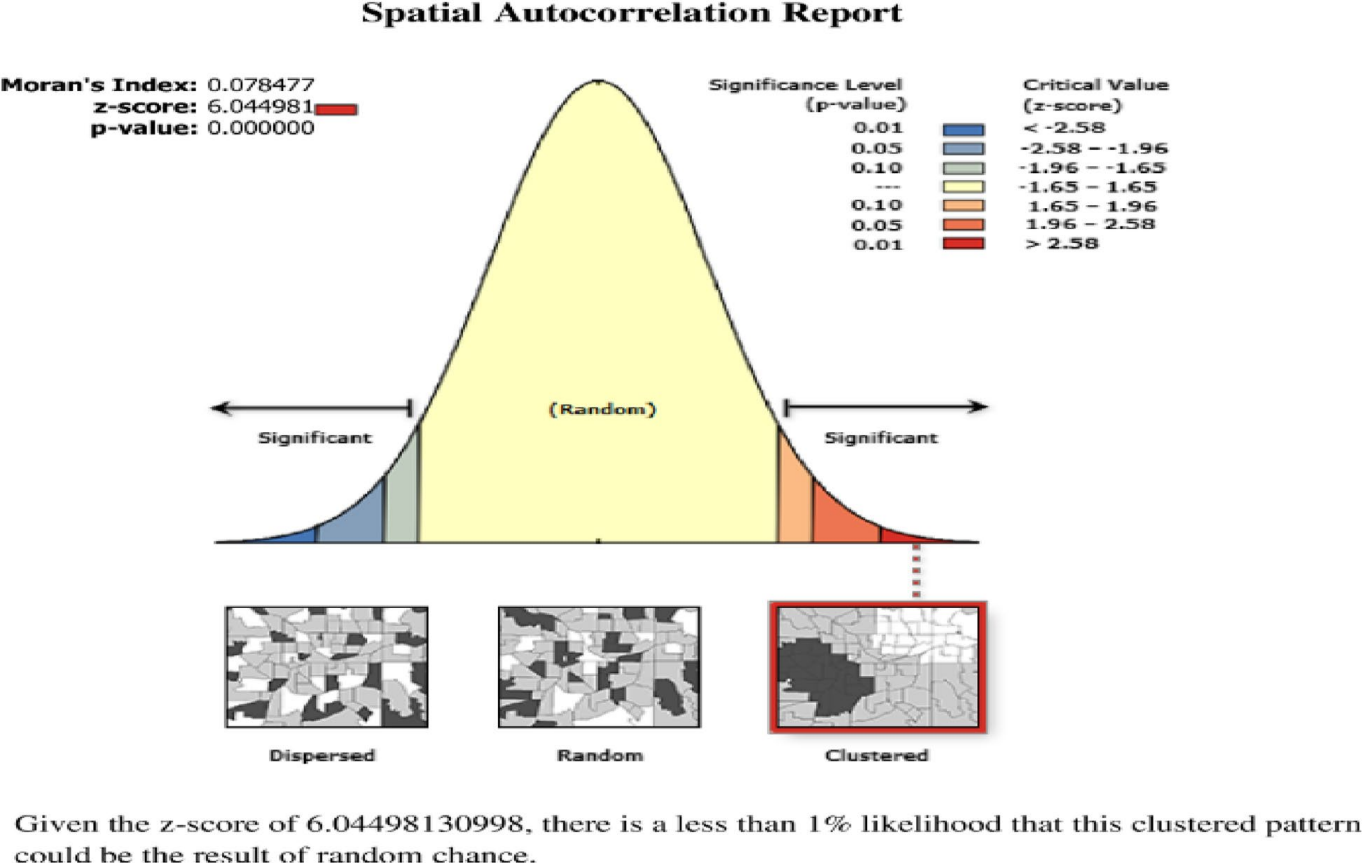
Spatial autocorrelation of modern contraceptive use among young women in Ethiopia: mini EDHS 2019

The spatial distribution of modern contraceptive use in Ethiopia was non-random (Moran’s I = 0.08, *p*-value < 0.01). The result showed that at the z-score of 6.044981, there was a less than 1% likelihood that such clustering pattern could be the result of random chance (Fig. 3).

### Hotspot analysis of modern contraceptive use

**Fig. 4:**
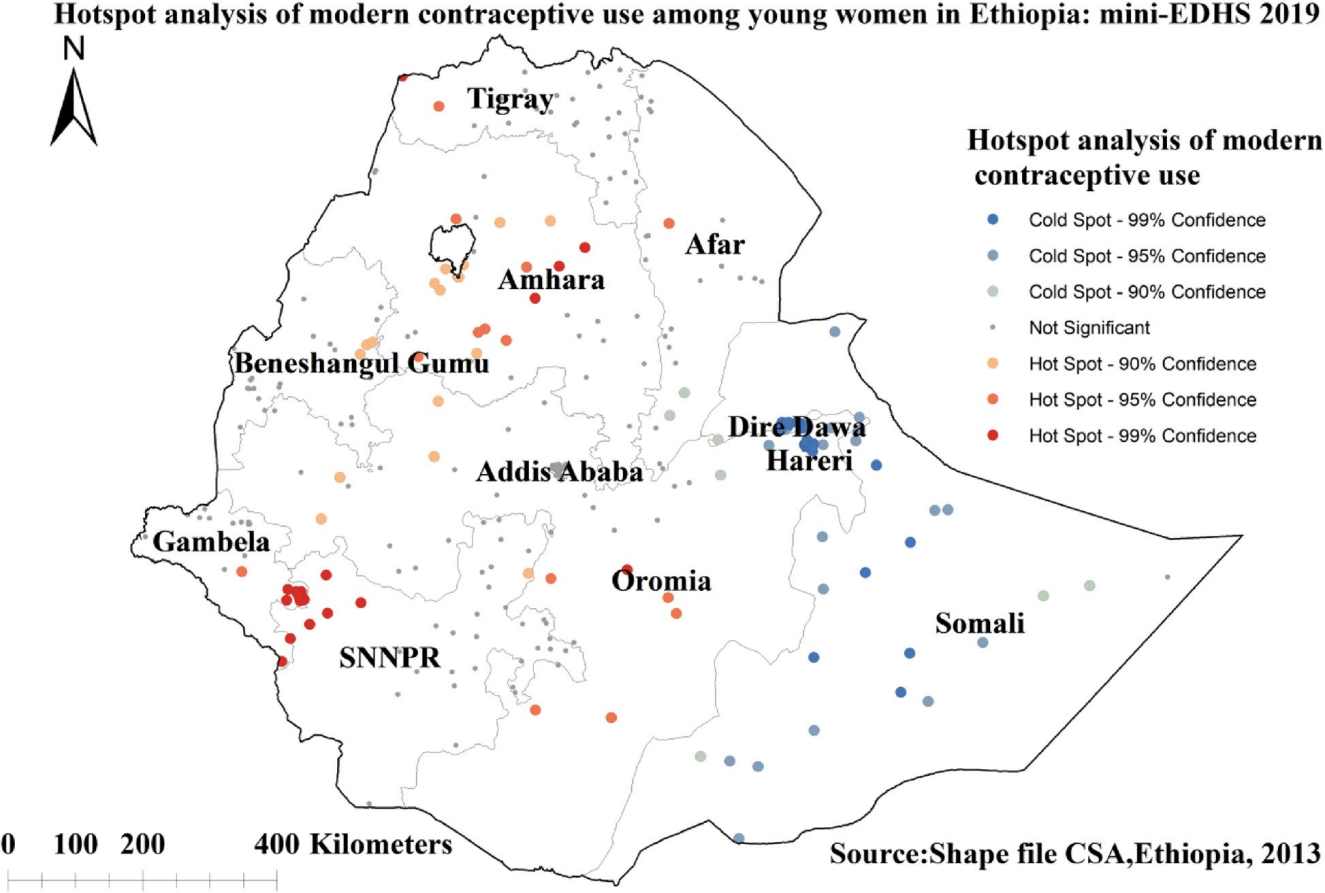
The hotspot analysis of modern contraceptive use among young women in Ethiopia: mini EDHS 2019

The Getis-Ord GI* statistical analysis identified the hotspot and cold spot areas of the modern contraceptive use across regions in Ethiopia. The blue color indicated the significant cold spot areas where a high proportion of women were not using modern contraceptives (clusters that had low modern contraceptive use) observed in most parts of Somali region, Dire dawa, and Harari cities. However, in the central and south-western Amhara, western and central Oromia, and North-western SNNPR, the red color indicated hotspot locations (clusters with high proportions of modern contraceptives use) (Fig. 4).

### Spatial interpolation or prediction of modern contraceptive use

**Fig. 5:**
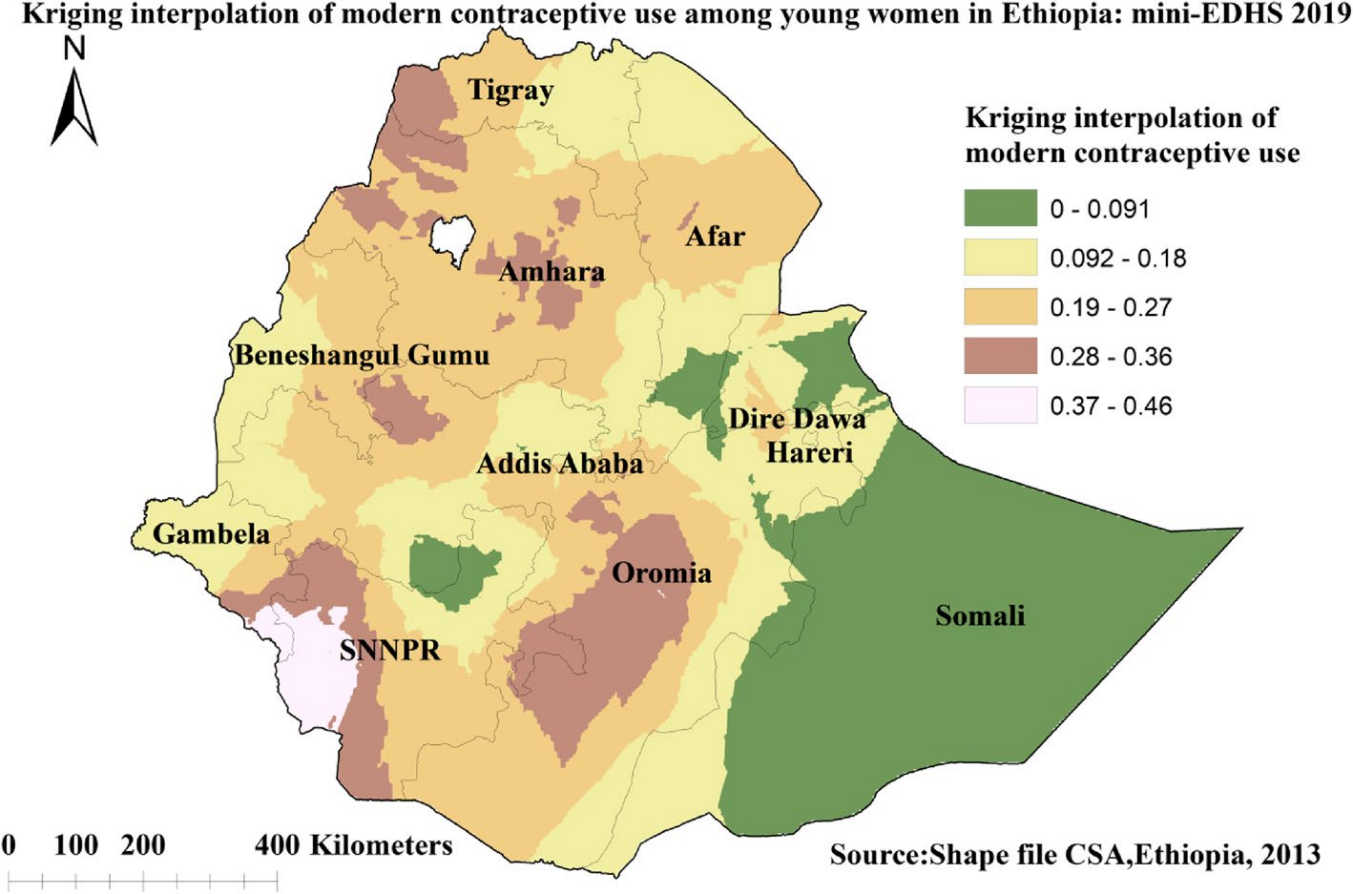
Spatial prediction of modern contraceptive use among young women in Ethiopia: mini EDHS 2019

The spatial interpolation technique shows the predicted proportion of modern contraceptive use for unsampled areas based on the sampled area in the Ethiopia. The ordinary Kriging method was used in describing the area map. The pink color indicates the predicted high proportion of modern contraceptive use in the country. Hence, if the area color changed from pink to green, it meant that the predicted modern contraceptive use decreases over the area. According to the prediction result, a high proportion of modern contraceptive use is located in western Tigray, western and central Amhara, some parts of Addis Ababa, central part of Oromia, and western and southern SNNPR. Whereas, a low proportion of modern contraceptive use is located in almost the entire Somali, central SNNPR, some parts of Dire Dawa and Harari as indicated in the green color (Fig. 5).

### Spatial scan statistical analysis

**Fig. 6:**
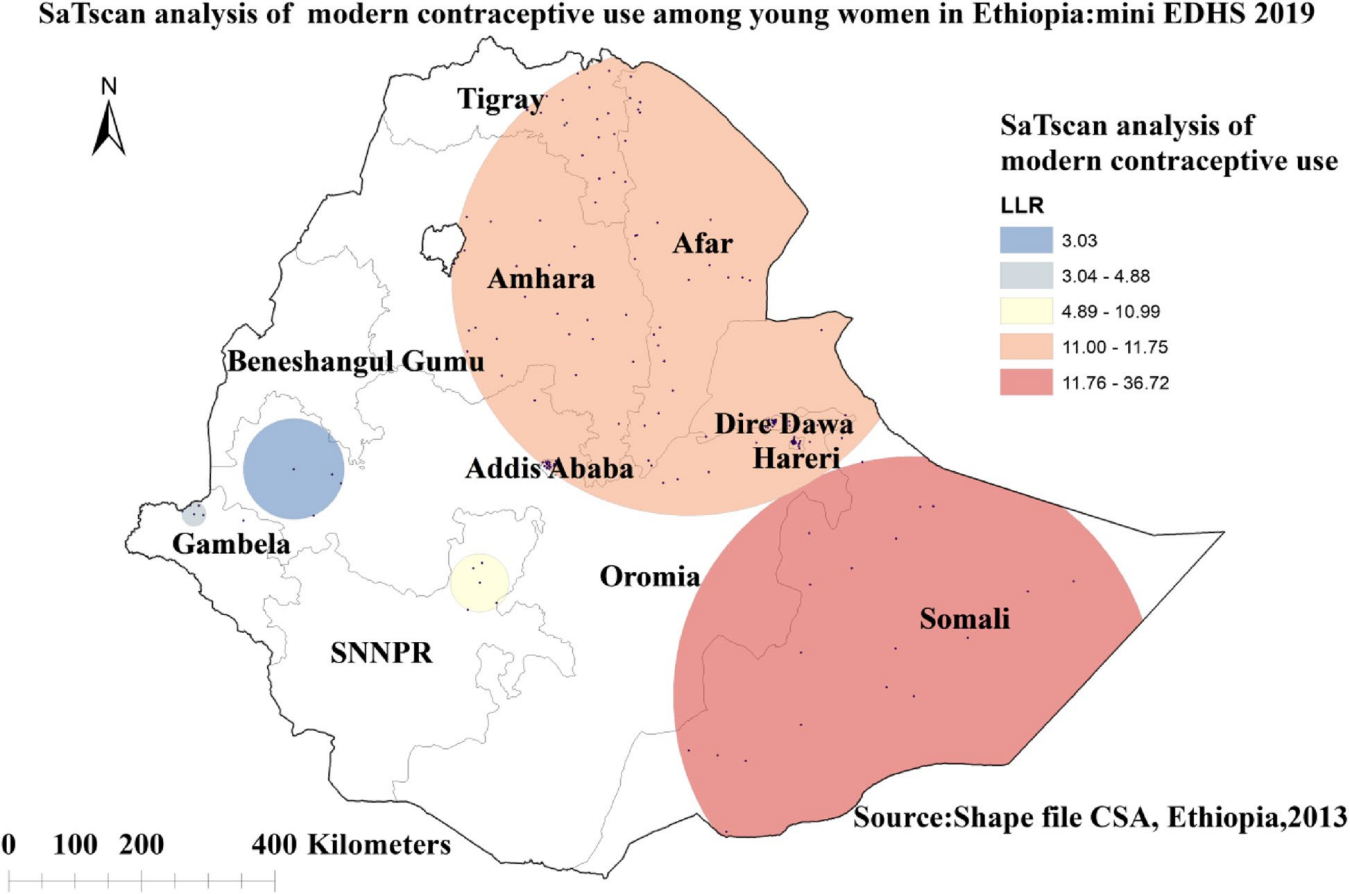
Sat Scan analysis of modern contraceptive use among young women in Ethiopia: mini EDHS 2019

A total of 166 most likely significant clusters of modern contraceptive non-users were detected in the spatial scan statistical analysis, indicating that women residing inside the spatial scan window were less likely to use modern contraceptives than women living outside the SaTScan window. Of these, 19 clusters of the areas were the most likely significant primary clusters. The most likely significant primary clusters were observed in the entire Somali, and some eastern parts of Oromia regions centered at 5.856584 N, 43.726017 E with a 360.74 km radius, a relative risk (RR) of 1.23, LLR of 36.7, with a *p*-value of less than 0.01. The second most significant clusters were located in South and eastern Tigray, the entire Afar, middle and eastern parts of Amhara, Dire Dawa, and Harari regions of Ethiopia centered at 11.531514 N, 40.697674 E with 355.89 km radius, RR = 1.08, LLR = 11.75, with *p*-value of 0.0025 (Fig. 6).

## Multilevel logistic regression analysis of modern contraceptive use among young women

### Random effect analysis

The ICC value in the null model was 0.29, indicating that 29% of the total variability in modern contraceptive use was attributable to the between-cluster variability, while about 71%% was attributable to individual differences. The MOR in the null model was 2.38, which indicates if we randomly pick a woman from two separate clusters, a woman with a higher probability of using modern contraceptive in the cluster had a 2.38 times higher probability of using it than a woman with lower likelihood of using the modern contraceptive in the cluster. The full model (Model III) was the best-fitted model for it has the highest log likelihood (-897) and the lowest deviance (1,794) value. The PCV in model III was 0.93, denoting that about 93% of the total variability in the modern contraceptive use was explained by the full model (Table 2).

**Table 2:** Multi-variable multilevel logistic regression analysis results of both individual-level and community-level factors associated with modern contraceptive use among young women in Ethiopia

**Variables**	**Categories**	**Null model**	**Model I** **AOR [95% CI]**	**Model II** **AOR** **[95% CI]**	**Model III** **AOR [95% CI]**
**Individual level factors**					
**Age in years**	15–19	Reff	0.76 (0.55, 1.04)		0.78 (0.57, 1.08)
	20–24		Reff		Reff
**Educational level**	no education		0.59 (0.30, 1.16)		0.73 (0.36, 1.44)
	primary		1.23 (0.70, 2.22)		1.27 (0.71, 2.27)
	secondary		1.58 (0.86, 2.92)		1.55 (0.84, 2.86)
	higher		Reff		Reff
**Wealth status**	poorest		0.46 (0.25, 0.85)		0.54 (0.26, 1.11)
	poorer		0.90 (0.50, 1.60)		0.85 (0.44, 1.66)
	middle		1.29 (0.7, 2.27)		1.16 (0.62, 2.18)
	richer		0.85 (0.52, 1.39)		0.76 (0.44, 1.30)
	richest		Reff		Reff
**Marital status**	married		17.94 (12.23, 26.27)		**18.5 (12.66, 27.27)**
	Not married		Reff		Reff
**Family size**	Less than 5		Reff		Reff
	5 and above		0.44 (0.33, 0.59)		**0.46 (0.34, 0.62)**
**Parity**	None		Reff		Reff
	One and above		4.79 (1.27, 18.08)		**4.82 (1.27, 18.32)**
**Number of living children**	None		Reff		Reff
	One and above		0.86 (0.23, 3.19)		0.75 (0.19, 2.80)
**Women gave birth in the past 3 years**	None		Reff		Reff
	One and above		0.75 (0.47, 1.18)		0.84 (0.53, 1.34)
**Son (s) present at home**	None		Reff		Reff
	One and above		1.35 (0.85, 2.16)		1.47 (0.91, 2.38)
**Daughter (s) present at home**	None		Reff		Reff
	One and above		2.27 (1.45, 3.55)		**2.45 (1.55, 3.86)**
**Household has TV**	Yes		2.20 (1.33, 3.62)		**2.39 (1.43, 3.99)**
	No		Reff		Reff
**Household has radio**	Yes		1.37 (1.01, 1.86)		**1.43 (1.05, 1.94)**
	No		Reff		Reff
**Community level factors**					
**Place of residence**	Urban			Reff	Reff
	Rural			0.82 (0.50, 1.34)	0.96 (0.48, 1.91)
**Region**	Tigray			1.79 (0.78, 4.10)	1.28 (0.43, 3.84)
	Afar			1.04 (0.27, 4.00)	0.34 (0.06, 1.80)
	Amhara			2.88 (1.32, 6.29)	2.81 (0.99, 7.94)
	Oromia			1.78 (0.83, 3.80)	0.99 (0.36, 2.73)
	Somali			0.09 (0.02, 0.37)	**0.05 (0.01, 0.32)**
	Benishangul			1.93 (0.60, 6.21)	1.22 (0.24, 6.13)
	SNNPR			2.25 (1.01, 5.04)	1.60 (0.54, 4.72)
	Gambela			2.68 (0.62, 11.58)	0.96 (0.13, 6.92)
	Harari			0.88 (0.11, 6.70)	0.42 (0.03, 6.63)
	Addis Ababa			Reff	Reff
	Dire Dawa			0.87 (0.22, 3.50)	0.53 (0.09, 3.23)
**Proportion of Muslims in the cluster**	High			1.07 (0.70, 1.62)	0.73 (0.42, 1.25)
	Low			Reff	Reff
**Community-level education**	High			0.74 (0.51, 1.06)	1.15 (0.70, 1.88)
	Low			Reff	Reff
**Community-level wealth**	High			1.16 (0.77, 1.3)	1.04 (0.58, 1.85)
	Low			Reff	Reff
**Random effect**					
	Variance	0.85	0.69	0.35	0.06
	ICC	0.29	0.24	0.20	0.17
	MOR	2.38	2.15	1.53	0.63
	PCV	Reff	0.19	0.59	0.93
**Model Comparison**					
	LLR	-1624	-925	-1599	897
	Deviance	3.248	1,850	3,198	1794

*ICC* Inter cluster correlation coefficient, *MOR* Median odds ratio, *PCV* proportional change in variance, *LLR* Log likelihood ratio, *AOR* adjusted odds ratiom, *CI* Confidence interval, *Reff* reference

### Fixed effect analysis

According to the multi-level multivariable regression analysis, married young women had higher odds of using modern contraceptives compared to their counterparts (AOR = 18.5; 95% CI: 12.66, 27.27). Also, the odds of modern contraceptive use among young women who were members of a family size above five was decreased by 54% (AOR = 0.46; 95% CI: 0.34, 0.62) compared to those who were from a family size of less than five. Again the odds of modern contraceptive use among young women who reside in Somali region was decreased by 95% (AOR = 0.05; 95% CI: 0.01, 0.32) compared to women living Addis Ababa. The present study also noted that a woman who gave 1 or more birth ever (parity) were nearly 5 times more likely to use modern contraceptive compared to a nulliparous woman (AOR = 4.82; 95% CI: 1.27, 18.32)**.** Moreover, the odds of using modern contraceptives among women who lived in a household where one or more daughters present was two and half times higher compared to women from a household with no daughter in the household (AOR = 2.45; 95% CI: 1.55, 3.86). The odds of using modern contraceptives among young women from a household which had TV or radio was (AOR = 2.39; 95%CI: 1.43, 3.99) and (AOR = 1.43; 95%CI: 1.05, 1.94), respectively (Table 2).

## Discussion

In this study, we assessed the spatial distribution of modern contraceptive use and the individual and community level factors affecting it among young women in Ethiopia using the 2019 mini-EDHS. In the analysis, it was found that there was considerable disparity across regions regarding modern contraceptive use among young women in Ethiopia.

Overall, the pooled prevalence of modern contraceptive use among young women was 17.23% (95% CI: 10.98, 23.47). The study also revealed a regional variation in the proportion of modern contraceptive use where a higher proportion of modern contraceptive use was located in the Amhara region while lower proportions observed in Somali region. The reason could be attributed to the variation in terms the contraceptive knowledge and attitude, educational status, access to family planning information, and cultural differences of study participants [20, 30]. Besides, the large increase in the use of modern contraceptive use in Amhara region could be attributed to the high number of family planning organizations and government’s focus in the region [31].

Compared to the previous studies from Ghana [32], Burkina Faso [33], Malawi [14], Senegal [22], Ethiopia (EDHS 2016), and other 20 African countries [8], the prevalence of modern contraceptive use among young women in our study was lower. The population difference, study setting, sample size, time difference of the surveys could be the possible reasons for the observed difference in the prevalence of modern contraceptives utilizations between our finding and across studies. For example, some of the aforementioned studies were limited only to married women where contraceptive utilization behaviour is higher among married women as reported in previous studies [34, 35]. Also, our study revealed that married women had higher odds of using modern contraceptives compared to the unmarried (AOR = 18.5; 95% CI: 12.66, 27.27). The possible justification for this is that married women had high likelihood to be pregnant and as a result, they usually prefer to use contraceptives to space births [36]. On the other hand, our study noted a higher prevalence of modern contraceptive use among young women compared to few studies in Zambia [37], Guinea [38], Western Nepal [19], Uganda [39]. The higher prevalence of modern contraceptive use in our study could be due to the survey year where our study is most recent and may be related availability of expanded services recently and could also be the countries difference in the investments of family planning initiatives by the non-governmental and governmental organizations.

In the spatial analysis, spatial autocorrelation, hotspot, and spatial scan statistical analysis were reported. The spatial autocorrelation statistic confirmed that the distribution of modern contraceptive use was clustered in some geographical areas. The hotspot analysis identified areas that had a low and high distribution of modern contraceptive use. The high proportions of modern contraceptive use (hotspots) areas were detected in the central and south-western Amhara, western and central Oromia, and North-western SNNPR regions. In agreement to this finding, the high rate of modern contraceptive use among married reproductive age women was observed in Amhara region, the SNNPR, and in some parts of Oromia region [31]. Conversely, the cold spot areas (lower proportion of modern contraceptive use) were observed in most parts of Somali region, Dire Dawa, and Harari cities. Similar geo-locations in modern contraceptive use was detected in previous DHS data based findings in Ethiopia where regions such as Somali, Afar, and Benishangul-Gumuz were cold spot areas for modern contraceptive use [31, 40]. The women’s level of awareness towards contraceptives, the diverse socio-cultural differences across regions, access to media and the decision making abilities of women have paramount importance for the utilizations of modern contraceptives [20, 21, 40]. Moreover, the increased proportion of the modern contraceptives use in the Amhara, Oromia, and the SNNPR regions, might be related to the availability and accessibility of infrastructures including family planning services through the coordinated collaboration among the government, non-governmental organizations and other stakeholders [41–44]. Thus, such kind of promising efforts to address the unmet need in family planning services is recommended to be expanded in the cold spot areas/regions (low rates of modern contraceptive use) identified in this study.

The SaTScan analysis identified 166 (19 primary and 147 secondary) most likely significant clusters of areas with low rate of modern contraceptive use across the study area, implying that young women living in those geographic clusters of areas had a lower chance of using modern contraceptives than women living outside the spatial scan window. The most likely significant primary clusters were observed in the entire Somali region, and some eastern parts of Oromia region whereas the secondary most significant clusters were located in South and eastern Tigray, the entire Afar, middle and eastern parts of Amhara, Dire Dawa, and Harari regions of Ethiopia (Fig. 6). In line with this, the fixed effect analysis of our data noted that the odds of modern contraceptive use among young women who reside in Somali region was decreased by 95% (AOR = 0.05; 95% CI: 0.01, 0.32) compared to women living in Addis Ababa. Such regional inequalities in the modern contraceptive uptake among women might be related to variations in the availability and accessibility of family planning services across the administrative regions of the country.

The current study has also assessed both individual and community level factors associated with the use of modern contraceptives among young women in Ethiopia. Accordingly, married women had higher odds of using modern contraceptives compared to the unmarried (AOR = 18.5; 95% CI: 12.66, 27.27). This association was in line with studies from Rwanda [34], Ghana [45], Malawi [14], Uganda [35]. There is an obvious reason that married women has a higher likelihood to become pregnant anytime and they would prefer to use contraceptives to prevent unintended pregnancy and to limit the number of children they need to have. Besides, married women are more likely to afford contraceptives compared to the unmarried perhaps due to partner support [45]. Moreover, evidences have noted that there is a customary pressure for a woman to give birth quickly after marriage [46, 47]. On the contrary, some studies reported that married women were less likely to use modern contraceptives than unmarried women [11, 32, 48, 49].

The present study also noted that a woman who gave 1 or more birth ever (parity) were nearly 5 times more likely to use modern contraceptive compared to a nulliparous woman (AOR = 4.82; 95% CI: 1.27, 18.32). In line with this finding, previous studies have noted a positive association between parity and use of modern contraceptives [13, 50–52]. Indeed, as parity increases, the desired number of children a woman planned to have may be achieved and thus the more likely a woman to rely on modern contraceptive methods to stop giving further birth and prevent unintended pregnancy [32].

The current study has also revealed that presence of television and/or radio in the household has a positive influence on the use of modern contraceptives among young women. Analogues to this finding, studies elsewhere reported such a positive association between access to media and use of modern contraceptives [10, 15, 18]. In fact, exposure of women to family planning information through radio, television, and newsletter increased the probability of using modern contraceptives [18, 51].

There are some notable strengths and limitations to the current study. As a strength, we used a combination of statistical methods (spatial analysis and multi-level logistics analysis) to explore the contextual and geographical factors in the use of modern contraceptives among young women (15–24 years), and key population group using the most recent DHS data-the 2019 mini EDHS. However, though the study focused on key individual and contextual risk factors, it has not addressed wider social and cultural environment contexts which may affect outcome. Besides, we have not included female autonomy, women attitudes toward family planning services use, partner/husband factors, community fertility norms, community level approval of family planning, road access and distance from health facility, which were found to affect the use of modern contraceptives in previous studies [51, 53–56] since these variables were not found in the data set.

## Conclusion

The utilisation of modern contraceptives was low and considerably varied across regions among young women in Ethiopia. Being married, family size, parity, presence of television and/or radio in the household, and living in Somali region were significant determinants of modern contraceptive use. Hence, family planning education programs should be broadcasted through mass media platforms, which will help improve modern contraceptive utilization among young women in Ethiopia.

In addition to the multilevel analysis, this study has identified the hot spot and cold spot areas to help the government in improving the provision of modern contraceptives, especially those areas with the low rates of modern contraception (Somali region). Taking in to account a geographic perspective on the distribution of contraceptive uptake and key factors identified in this study would be vital for efficient resource allocation, targeted interventions, and informed decision-making, and monitoring and evaluation purposes.

## Data Availability

Data are available online in a public, open-access repository (www.measuredhs.com/data).

## Abbreviations

AOR: Adjusted Odds Ratio
DHS: Demographic and Health Surveys
EMDHS: Ethiopian mini Demographic and Health Survey
EAs: Enumeration Areas
GIS: Geographic Information System,
HSTP-I: Health Sector Transformation Plan I
ICC: Intra-cluster correlation coefficient
MOR: Median odds ratio
PCV: A proportional change in variance
IR: Personal record
WHO: World Health Organization
